# Differential Expression of MicroRNAs in Adipose Tissue after Long-Term High-Fat Diet-Induced Obesity in Mice

**DOI:** 10.1371/journal.pone.0034872

**Published:** 2012-04-04

**Authors:** Dionysios V. Chartoumpekis, Apostolos Zaravinos, Panos G. Ziros, Ralitsa P. Iskrenova, Agathoklis I. Psyrogiannis, Venetsana E. Kyriazopoulou, Ioannis G. Habeos

**Affiliations:** 1 Department of Internal Medicine, Division of Endocrinology, Medical School, University of Patras, Patras, Greece; 2 Laboratory of Clinical Virology, Medical School, University of Crete, Heraklion, Greece; The University of Hong Kong, Hong Kong

## Abstract

Obesity is a major health concern worldwide which is associated with increased risk of chronic diseases such as metabolic syndrome, cardiovascular disease and cancer. The elucidation of the molecular mechanisms involved in adipogenesis and obesogenesis is of essential importance as it could lead to the identification of novel biomarkers and therapeutic targets for the development of anti-obesity drugs. MicroRNAs (miRNAs) have been shown to play regulatory roles in several biological processes. They have become a growing research field and consist of promising pharmaceutical targets in various fields such as cancer, metabolism, etc. The present study investigated the possible implication of miRNAs in adipose tissue during the development of obesity using as a model the C57BLJ6 mice fed a high-fat diet.

C57BLJ6 wild type male mice were fed either a standard (SD) or a high-fat diet (HFD) for 5 months. Total RNA was prepared from white adipose tissue and was used for microRNA profiling and qPCR.

Twenty-two of the most differentially expressed miRNAs, as identified by the microRNA profiling were validated using qPCR. The results of the present study confirmed previous results. The up-regulation of mmu-miR-222 and the down-regulation of mmu-miR-200b, mmu-miR-200c, mmu-miR-204, mmu-miR-30a*, mmu-miR-193, mmu-miR-378 and mmu-miR-30e* after HFD feeding has also been previously reported. On the other hand, we show for the first time the up-regulation of mmu-miR-342-3p, mmu-miR-142-3p, mmu-miR-142-5p, mmu-miR-21, mmu-miR-146a, mmu-miR-146b, mmu-miR-379 and the down-regulation of mmu-miR-122, mmu-miR-133b, mmu-miR-1, mmu-miR-30a*, mmu-miR-192 and mmu-miR-203 during the development of obesity. However, future studies are warranted in order to understand the exact role that miRNAs play in adipogenesis and obesity.

## Introduction

Obesity has emerged as an epidemic in modern societies [1] and has detrimental effects on the quality of life and the life expectancy of people as it increases the risk of cardiovascular disease, metabolic syndrome and cancer [2], [3].

Obesity is characterized by an excess of white adipose tissue mass. Two types of adipose tissue are found in mammals, white and brown adipose tissue (WAT and BAT). WAT is found below the skin (subcutaneous WAT) and inside the abdomen (abdominal WAT) while BAT is found in the interscapular region and axillae in rodents and in upper chest and neck in humans. The WAT has been characterized as an endocrine organ itself [4]. It produces endocrine acting peptides such as leptin and is metabolically important as WAT excess is associated with metabolic syndrome [5], [6], [7]. BAT is specialized in energy expenditure and its activation can counteract obesity [8], [9], [10]. Despite the research that has been made in the field of obesity and adipocyte differentiation, the exact mechanisms involved in these processes remain to be elucidated.

The non-coding microRNAs (miRNAs) which were discovered in the last decade [11], [12] and are currently known to post-transcriptionally regulate genes that are involved in various biological processes (cell growth, apoptosis, metabolism) [13], [14], have come in the spotlight of research in obesity and metabolic syndrome [15], [16], [17]. The existing studies on miRNAs and obesity indicate that these molecules may play an important role in the development of obesity and can possibly be promising targets for future therapeutic interventions.

A popular method to identify miRNAs that may play a role in adipogenesis and obesogenesis is the performance of miRNA arrays in animal (mouse) models of obesity (genetically or diet-induced) and in preadipocyte cell-culture models that are induced to differentiate into adipocytes [18]. In this way, miRNAs that change significantly during these processes are identified and then they can be checked with reference to their exact role in obesity or adipocyte differentiation. The majority of the existing studies with miRNA arrays in obesity and adipogenesis models [17], [19], [20] use the C57BLJ6 mouse after HFD feeding for 3 months as a model.

In the present study we investigated the changes that occur in miRNA expression in white adipose tissue after long-term (5 months) high fat diet (HFD) feeding in C57BLJ6 mice, using miRNA profiling and qPCR. We verified previously reported changes in miRNA expression. Moreover, we identified for the first time some new miRNAs that change significantly during long-term diet-induced obesity. These miRNAs could be proved to be valuable pharmaceutical targets for anti-obesity treatment.

## Materials and Methods

### Ethics statement

All animal procedures were approved by the institutional review board of the University of Patras Medical School (approval ID 15/2008) and were in accordance with EC Directive 86/609/EEC.

### Mice

The C57BL6J wild type male mice (8 weeks old) were used. Eight mice were fed a standard diet (SD) (10 kcal% fat) and other eight were fed an HFD (60 kcal% fat; Research Diets, New Brunswick, NJ) for 5 months. Mice were housed in the animal facility of the University of Patras Medical School in temperature-, light- and humidity-controlled rooms with a 12-h light/dark cycle. Weight measurements were performed on a weekly basis to confirm that the HFD-fed mice gain more weight than the SD-fed ones. Evaluation of blood glucose levels and of insulin sensitivity was performed as described previously [21] at the beginning and in the end of the experiment, in both groups, so as to confirm that the HFD-fed mice developed an obese and insulin resistant phenotype.

### White adipose tissue total RNA extraction

White adipose tissue (WAT) was excised from mice from epididymal fat pads and was submerged immediately in RNA later solution (Ambion, Foster City, CA). Total RNA was isolated from WAT from SD- or HFD-fed mice using the TRIzol reagent (Invitrogen, Carlsbad, CA), following the manufacturer's instructions. The individual RNA samples from SD- or HFD-fed mice were pooled for microarray experiments. The Reverse-Transcription PCR was performed in individual samples. The quality of the total RNA for microRNA profiling was assessed using an Agilent 2100 Bioanalyzer profile (Agilent Technologies, Santa Clara, CA). Total RNA concentration was measured with a Nanodrop instrument, calculating the ratio A_260_/A_280_.

### microRNA profiling

One µg total RNA from sample and reference were labeled with Hy3™ and Hy5™ fluorescent label, respectively, using the miRCURY™ LNA Array power labeling kit (Exiqon,Denmark) following the procedure described by the manufacturer. The Hy3™-labeled samples and the Hy5™-labeled sample were mixed pair-wise and hybridized to the miRCURY™ LNA array version 10.0 (Exiqon,Denmark ), which contains capture probes targeting all miRNAs for all species registered in the miRBASE version 11.0 at the Sanger Institute. The hybridization was performed according to the miRCURY™ LNA array manual using a Tecan HS4800 hybridization station (Tecan, Austria). After hybridization, the microarray slides were scanned and stored in an ozone free environment (ozone level below 2.0 ppb) in order to prevent potential bleaching of the fluorescent dyes. The miRCURY™ LNA array microarray slides were scanned using the Agilent G2565BA Microarray Scanner System (Agilent Technologies, Inc., USA) and the image analysis was carried out using the ImaGene 8.0 software (BioDiscovery, Inc., USA).

### Microarray Data Filtering, Background Correction and normalization

Filtering was performed based on the signal intensity. Spots with no signal above the background (flags 1 and 2) were detected and removed. Background correction was performed to remove non-biological contributions (“background”) to the measured signal, by subtracting the median global background from the median local background from the signal intensity. A threshold of 2 was set as cut-off, meaning that spot intensity for at least one channel should be twice as much as that of the background. Normalization was performed using the Lowess regression algorithm, in order to remove certain systematic biases from microarray data, such as dye effects or intensity dependence (Fig. S1 and Fig. S2). The positive effect from normalization is illustrated on each slide sheet with an M-A plot before and after normalization (Fig. S3). After normalization the spots appeared symmetrically scattered around the horizontal line (M = 0). Normalized data were extracted, pre-processed and sorted with Microsoft Excel ®.

### Slide quality check using spike-in controls

Spike-in CV values were calculated between 32 replicates for each of the spike-in controls on each slide. CVs did not exceed 30%, thus reflecting lack of spatial effects on the array (smear across the slide) or misplacement of the grid for annotating the spots.

### Clustering

We performed clustering of the miRNAs using four methods: Hierarchical clustering (HCL), k-means clustering, Principle Components Analysis (PCA) and TIGR clustering (terrain classification) using 3-dimensional representation of the clustering results.

### GEO accession numbers

Our miRNA profiling data were MIAME compliant and deposited at the Gene Expression Omnibus (GEO) repository, with accession numbers from GSM802367 through GSM802369 (http://www.ncbi.nlm.nih.gov/geo/query/acc.cgi?token=btghlsqqmoessvc&acc=GSE32419).

### Reverse-Transcription (RT) and qPCR validation

The microRNA in the total RNA sample was converted to cDNA by reverse transcription using the miRCURY™ LNA microRNA PCR system First-strand cDNA synthesis kit and a microRNA-specific primer. The cDNA was amplified by real-time PCR using SYBR® Green master mix and LNA™ microRNA-specific primers on a Step One Plus Instrument (Applied Biosystems, Foster City, CA) using the designated PCR optical tubes. The following 22 murine microRNAs were selected for qPCR validation of their expression: mmu-miR-1, mmu-miR-21, mmu-miR-30a*, mmu-miR-30e*, mmu-miR-122, mmu-miR-130a, mmu-miR-133b, mmu-miR-141, mmu-miR-142-3p, mmu-miR-142-5p, mmu-miR-146a, mmu-miR-146b, mmu-miR-192, mmu-miR-193a-3p, mmu-miR-200b, mmu-miR-200c, mmu-miR-203, mmu-miR-204, mmu-miR-222, mmu-miR-342-3p, mmu-miR-378 and mmu-miR-379. All runs included no template and RT-minus controls. A 5-fold serial dilution of pooled cDNA samples was generated for each assay, in order to calculate the PCR efficiency. All reactions were performed in triplicates. Before proceeding with data normalization, a technical quality assessment was performed based on results of the melting curve, serial dilution curve and no-template-controls. The stability and ranking of the endogenous controls was also calculated with the SLqPCR algorithim. For every endogenous control gene, the pair-wise variation with all other endogenous controls was determined as a gene stability measurement M. An M value below 1.5 was recommended and genes with expression stability above 1.5 were considered unstable across the samples and unsuitable for endogenous controls in this experiment. U6 snRNA, mmu-191, mmu-423-5p, mmu-361 and mmu-103 were considered acceptable and used for normalization [22]. The gene 5S rRNA was unstable across the samples and therefore excluded from the normalization (Fig. S4). Relative expression was performed using the ΔΔCt method [23], [24]. Data were standardized by log_2_ transformation.

### Gene Ontology (GO) and enrichment analysis

Gene Ontology (GO) analysis is essential in the deduction of conclusions from microarray data. GO is a database with curated annotations for known genes i.e. gene biological processes, molecular functions and cellular components. Firstly, using the miRWalk software [25] the validated target genes of the confirmed differentially expressed miRNAs were identified. Then, GO analysis was performed for the validated miRNA target genes, using the Genesis 1.7.2 software [26] and the WebGestalt web-tool (http://bioinfo.vanderbilt.edu/webgestalt) [27]). All gene definitions and functions were based on the National Institute of Health databases (http://www.ncbi.nlm.nih.gov/sites/entrez/). Further enrichment analysis for the validated miRNA targets was also investigated, using the WebGestalt web-tool (http://bioinfo.vanderbilt.edu/webgestalt) [27]. The hypergeometric test, with Bonferroni correction was used for enrichment evaluation analysis. The R function adjP was used in order to adjust the nominal p values of the large number of categories at the same time. The significance level for the adjusted p-value was set at 0.01 and the number of minimum genes for a category was set at 2.

### Statistical analysis

Normality of the data distribution was checked by Kolmogorov-Smirnov test. Differences in the expression levels between SD- and HFD-fed mice in WAT were explored using the Mann-Whitney U test. Numerical values are expressed as the mean±standard deviation. Statistical significance was set at the 95% level (p<0.05). In weight and blood glucose measurements in mice, as well as in qPCR, the Student t-test or the one-way ANOVA followed by Tukey's test was used for statistical analysis. These data were expressed as the mean±SEM or mean±standard deviation, as described in the corresponding figure legends. Statistical significance was set at the 95% level (p<0.05). The statistical package GraphPad Prism was used (GraphPad Software, La Jolla, CA).

## Results

In the present study, we examined the expression levels of 530 mmu-miRNAs in adipose tissue received from SD-fed mice and compared them with the corresponding expression in adipose tissue from HFD-fed mice for a 5-month period.

### Evaluation of the obese and insulin resistant phenotype in mice after 5 months on HFD

At the beginning of the experiment, mice were age- and weight-matched, and thus had no statistically significant differences in their weights (Fig. S5). After 5 months on HFD or SD, the HFD-fed mice were significantly heavier (p<0.0001) than the SD-fed mice, as expected (Fig. S5). Moreover, although the two mouse groups did not have any differences in glucose tolerance and insulin resistance at the beginning of the experiment, the HFD-fed mice developed a significantly less glucose tolerant and more insulin resistant phenotype than the SD-fed mice after 5 months on the diet, as revealed by the IPGTT (Intraperitoneal Glucose Tolerance Test) and by the IPITT (Intraperitoneal Insulin Tolerance Test), correspondingly (Fig. S6).

### MicroRNA profiling

**Figure 1:**
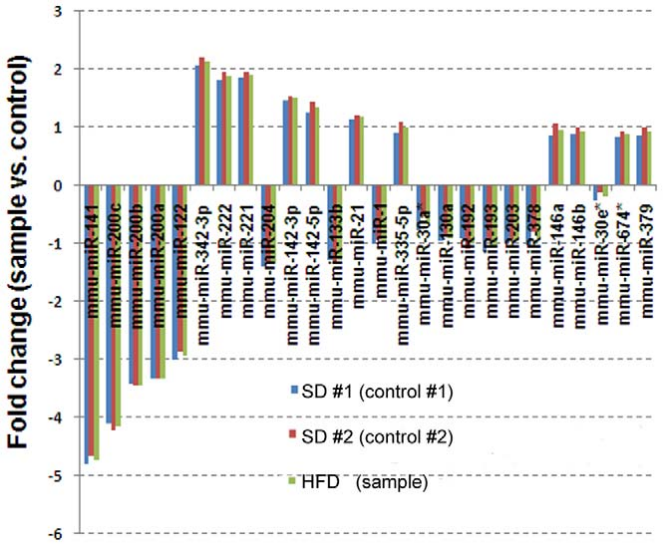
Differentially expressed miRNAs between control mice (standard diet) and sample mice (high-fat diet) mice. The SD #1 and #2 indicate technical replicates. The diagram shows the top ranking 26 differentially expressed miRNAs based on the comparison samples versus control. Each bar represents the fold change between the sample mice versus the control mouse. Since slide no. 3 is a dye swap (control vs. samples) the values have been reversed to fit slides no. 1 and 2. A fold change >1 indicates up-regulation after HFD feeding, whereas a fold change <1 indicates down-regulation after HFD feeding. SD; standard diet, HFD; high-fat diet.

Based on our miRCURY™ LNA array microarray data, we identified 26 differentially expressed miRNAs in adipose tissue between SD and HFD mice. The following miRNAs were found to be up-regulated in WAT after HFD feeding: mmu-miR-342-3p, mmu-miR-222, mmu-miR-221, mmu-miR-142-3p, mmu-miR-142-5p, mmu-miR-21, mmu-miR-335-5p, mmu-miR-146a, mmu-miR-146b, mmu-miR-647* and mmu-miR-379. On the contrary, the following miRNAs were down-regulated in WAT after HFD feeding: mmu-miR-141, mmu-miR-200a, mmu-miR-200b, mmu-miR-200c, mmu-miR-122, mmu-miR-204, mmu-miR-133b, mmu-miR-1, mmu-miR-30a*, mmu-miR-130a, mmu-miR-192, mmu-miR-193a-3p, mmu-miR-203, mmu-miR-378 and mmu-miR-30e*. The log_2_ fold change between SD and HFD mice is depicted in Figure 1.

### Clustering

**Figure 2:**
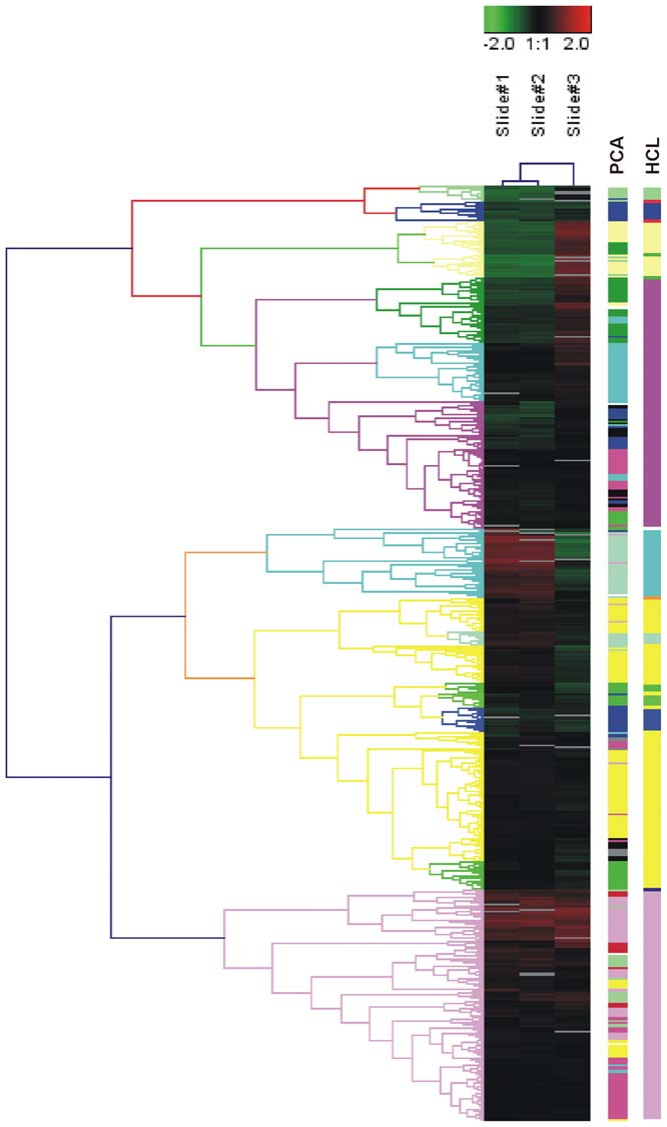
Unsupervised hierarchical cluster analysis diagram based on 529 probe sets with highest variation in SD vs. HFD mice. Color saturation is directly proportional to measured expression ratio magnitude. Rows represent individual probe set. Columns represent experimental sample. Red bars indicate high expression. Green bars indicate low expression. Slides 1 and 2: control (SD) mice, slide3: sample (HFD) mice. SD; standard diet. HFD; high-fat diet.

We performed Hierarchical clustering using Average Linkage Clustering and Euclidian Distance. The condensed tree and cluster image are depicted in Figure 2. Two big branches could be noticed, and each one was composed of smaller ones. The first branch (5 miRNA groups) contained mmu-miRNAs which were under-expressed in the SD vs. the HFD mice. The second branch was composed of 2 big groups, partitioned into 4 sub-clusters. On the contrary, the first group of the second big branch contained miRNAs which were over-expressed in the SD vs. the HFD mice. Finally, the second group of the second big branch contained miRNAs which were over-expressed both in SD and HFD mice. Each group is distinct by a different coloring.

**Figure 3:**
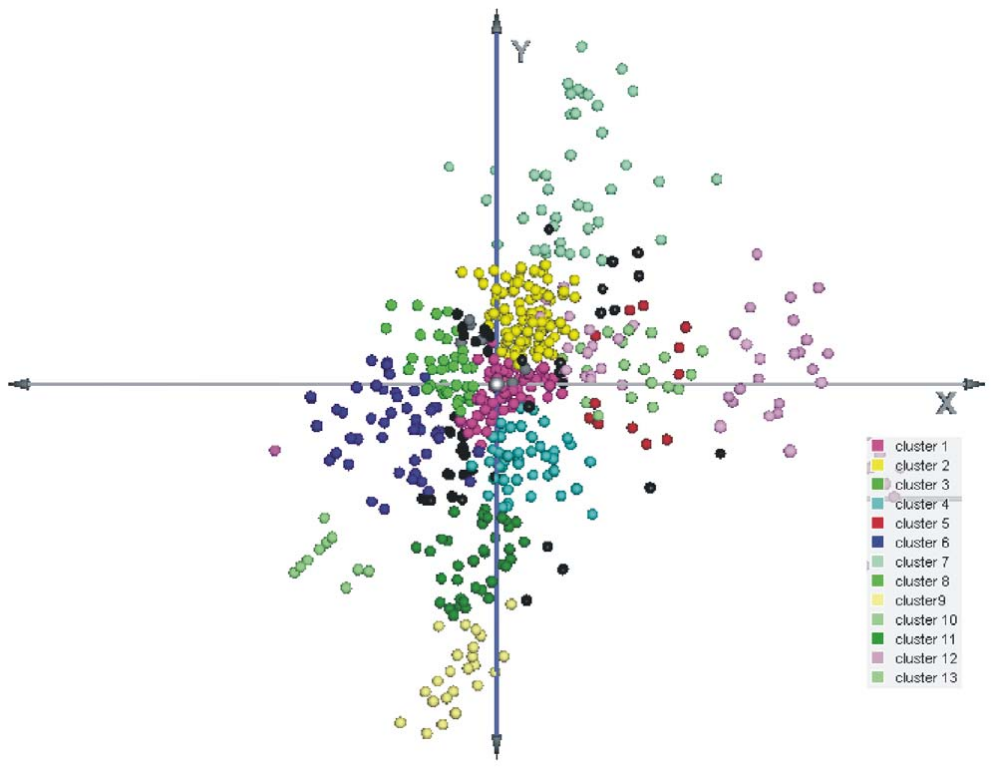
Three-dimensional PCA clustering. The observed samples were represented as a linear combination of the principal components with associated gene scores. The three-dimensional PCA clustering revealed 13 miRNA clusters.

**Figure 4:**
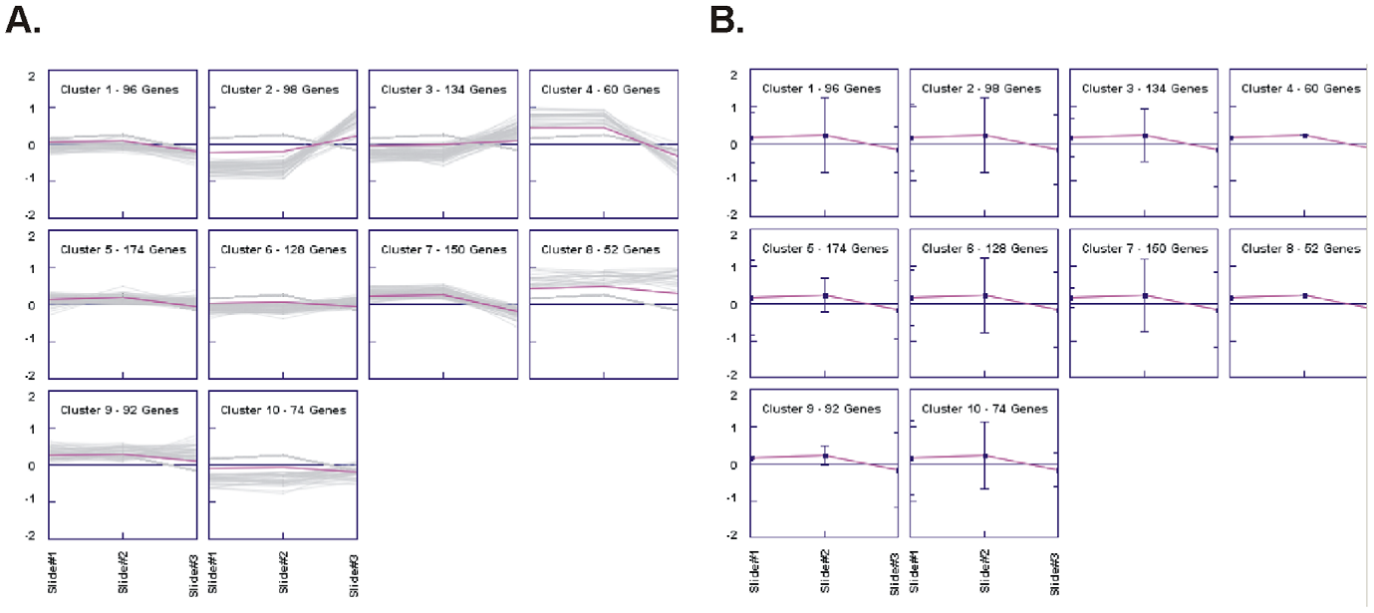
K-means clustering identified 10 clusters. This non-hierarchial method initially takes the number of components of the population equal to the final required number of clusters. In this step itself the final required number of clusters is chosen such that the points are mutually farthest apart. Next, it examines each component in the population and assigns it to one of the clusters depending on the minimum distance. The centroid's position is recalculated everytime a component is added to the cluster and this continues until all the components are grouped into the final required number of clusters. A. K-means clustering of profiles and B. Centroids.

Similar clustering was performed on the principal components (PCs) outputted by Principal Component Analysis (PCA) on the same original data (where the gene expression measurements are the variables and the samples are the observations). In the PCA method, the observed samples were represented as a linear combination of the PCs with associated gene scores. PCA showed an even larger number of miRNA groups. Thirteen clusters could be observed in a three-dimensional clustering (Fig. 3). The clustering results obtained by HCL and PCA are depicted side-by-side in Figure 2 for an immediate comparison between them. K-means clustering identified 10 clusters (Fig. 4). The number of genes in each cluster, the (%) share of genes in each cluster, the average distance from the cluster mean, the next neighbor variance, as well as the within cluster variance, are depicted in detail, in Table 1.

**Table 1:** K-means clustering details.

Cluster	Number of genes in cluster	Share of genes in cluster	Average distance from gene cluster	Next nearest neighbor variance	Within cluster variance
**Cluster 1**	96	18%	0.00666	0.05622	0.00510
**Cluster 2**	98	19%	0.02696	0.03822	0.01278
**Cluster 3**	134	25%	0.02221	0.06643	0.01069
**Cluster 4**	60	11%	1.49754E-7	0.05323	0.01152
**Cluster 5**	174	33%	0.00801	0.05567	0.00541
**Cluster 6**	128	24%	0.01260	0.06942	0.00403
**Cluster 7**	150	28%	0.00159	0.06104	0.00644
**Cluster 8**	52	10%	1.2731E-7	0.01901	0.01001
**Cluster 9**	92	17%	0.01223	0.03656	0.00835
**Cluster 10**	74	14%	0.01900	0.04451	0.01046

**Figure 5:**
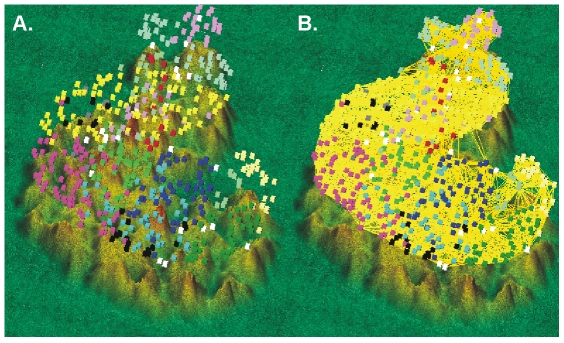
Terrain (map) analysis, depicting the gene clusters, as well as the links among the clustered genes. A. Each miRNA cluster is depicted with a different color. B. The correlations among the various miRNAs are depicted with lines.

**Figure 6:**
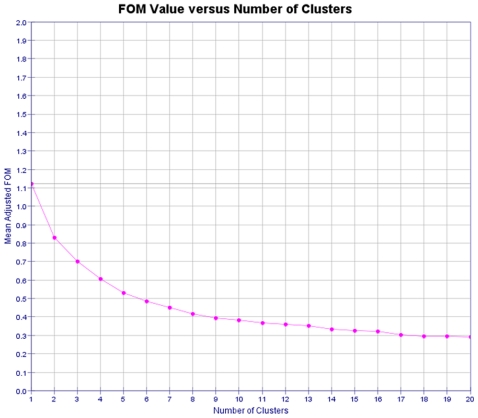
Figure of merit (FOM) vs. no. of clusters graph for the k-means cluster algorithm. A figure of merit is an estimate of the predictive power of a clustering algorithm. The lower the adjusted FOM value is, the higher the predictive power of the algorithm. The value of the adjusted FOM for the k-means run decreases steeply until the number of clusters reaches 7, after which it levels out. This suggests that, for this data set, k-means performs optimally for 7 clusters and that any additional clusters produced will not add to the predictive value of the algorithm.

A three-dimensional TIGR clustering analysis was also performed. The terrain (map) analysis, depicting the gene clusters, as well as the links among the clustered genes, is depicted in Figure 5. The predictive power of the K-means algorithm was estimated using a figure of merit (FOM). A FOM value vs. number of clusters was computed by removing each sample in turn from the data set, clustering genes based on the remaining data, and calculating the fit of the withheld sample to the clustering pattern obtained from the other samples (Fig. 6).

### qPCR validation of expression

**Figure 7:**
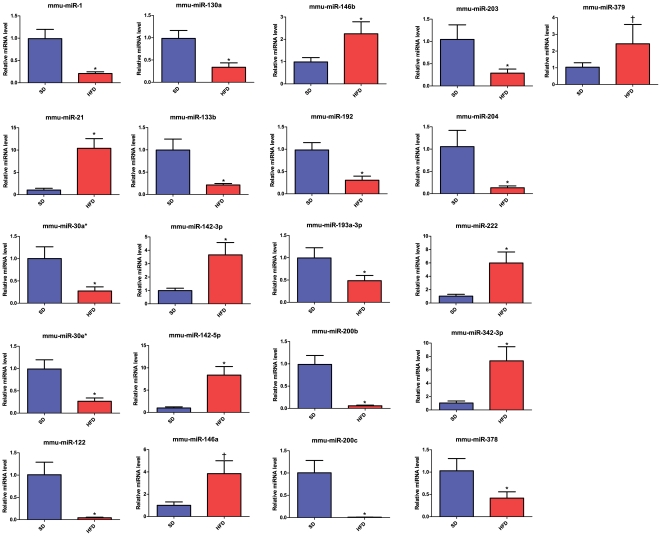
miRNA relative expression in mice fed a standard or a high-fat diet for 5 months. The miRNA levels were measured by quantitative RT-PCR in white adipose tissue from mice fed a standard or a high-fat diet for 5 months (n = 8 for each diet type). The RT-PCR was performed in triplicate wells for each individual sample. Bars show means±standard deviation. * p<0.0001, † p<0.001. SD; standard diet, HFD; high-fat diet.

**Figure 8:**
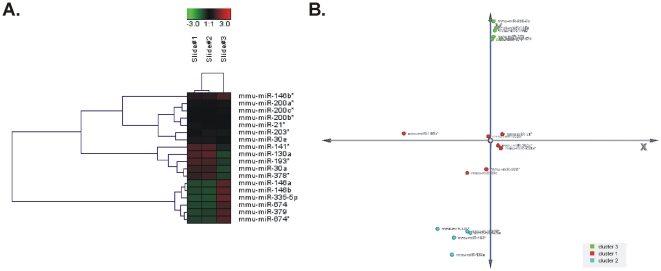
Hierarchical clustering (HCL) and Principal Component Analysis (PCA). HCL (A) and PCA analysis (B) for 18 mmu-miRs validated by qPCR. The clustering results were similar to those acquired by the miRNA profiling.

Twenty-two of the most differentially expressed miRNAs after HFD feeding, as revealed by our microRNA profiling, were also tested by qPCR. After normalizing the expression levels with the corresponding geometric mean value of the reference genes U6 snRNA, mmu-miR-191, mmu-miR-423-5p, mmu-miR-361 and mmu-miR-103, the samples were checked for outliers to be excluded. The qPCR verified the expression profile of the 22 DE miRNAs. The fold changes were calculated using the ΔΔCt comparative quantification method. Data were corrected for assay specific PCR efficiency (microRNA or endogenous control) and normalized with a normalization factor based on the five validated endogenous controls. Mmu-miR-141 was not detected in the SD mice (control), hence no fold change was calculated. The relative miRNA expression levels for each miRNA are depicted in Figure 7. Moreover, we performed HCL and PCA analysis for the qPCR validation data of 18 miRNAs (Fig. 8). The clustering results were similar to those acquired by the microarray data.

### Correlation between microrray and qPCR results

**Figure 9:**
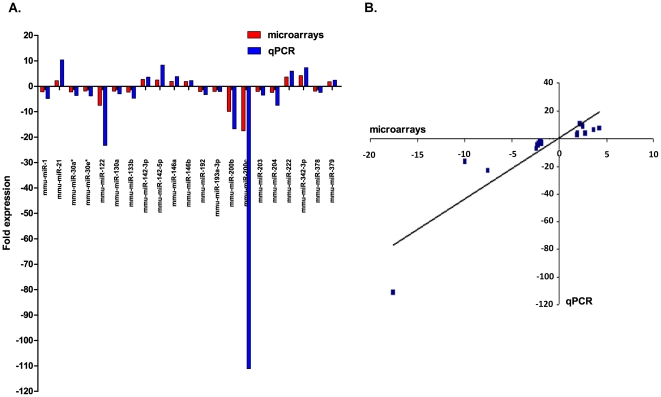
Correlation between microarrays and qPCR. A. The fold changes were calculated using the ΔΔCt comparative quantification method. Mmu-miR-141 was not detected in “control mice” (SD), hence no fold change was calculated. We identified an agreement between the microarrays and the qPCR results. B. Scatterplot and Correlation between qPCR and microarrays (Pearson's correlation = 0.888).

We identified an agreement between the microarray and the qPCR results, showing the relative expression of array data compared to the relative expression of qPCR data (Fig. 9A). The relative expressions were based on three technical replicates. The Pearson correlation value between microarray and qPCR data was 0.888 (Fig. 9B).

### Gene Ontology (GO)

**Figure 10:**
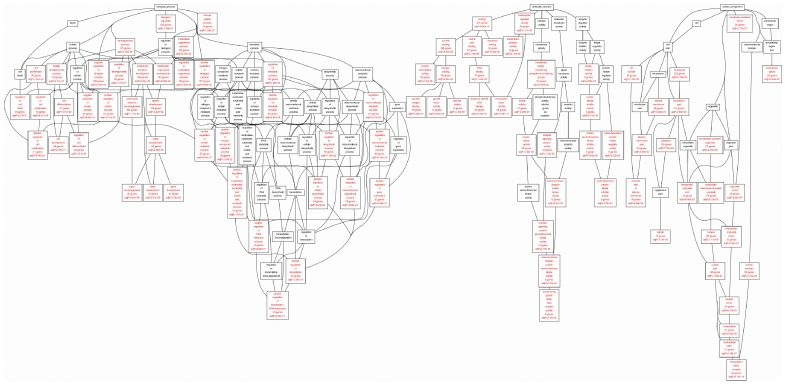
GO terms annotation of the validated target genes of the up-regulated mmu-miRNAs after HFD feeding. The most significant functions of the enriched miRNAs are highlighted in red color.

**Figure 11:**
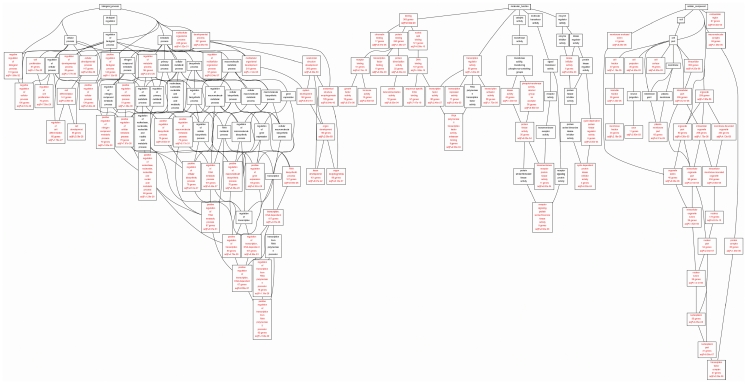
GO terms annotation of the validated target genes of the down-regulated mmu-miRNAs after HFD feeding. The most significant functions of the enriched miRNAs are highlighted in red color.

GO analysis was performed in order to approach the functionality of the target genes of the studied miRNAs. Firstly, the validated target genes of the differentially expressed miRNAs were identified using the miRWalk algorithms shown in Table S1, which is divided into the up- and the down-regulated miRNAs. GO analysis was initially performed for the target genes of the up-regulated miRNAs and then for the down-regulated ones. The genes were initially separated into three groups, based on their molecular function, biological process and cellular component. The enriched processes of the up-regulated miRNA targets, along with their statistical significance, are depicted in Figure 10. Cell differentiation (adjP = 1.41e-30) and metabolic processes (regulation of metabolic process, adjP = 1.85e-25) are among the most significant biological processes related to the development of obesity. Table S2 includes the genes that participate in all the depicted enriched processes. Figure 11 exhibits the enriched processes for the target genes of the down-regulated miRNAs. Again the enrichment of processes related to obesity, such as regulation of metabolic process (adjP = 3.21e-25) or cell differentiation (adjP = 4.89e-35), is high. Table S3 depicts the genes that participate in all the depicted enriched processes.

## Discussion

The current study investigated the changes that occur in miRNA expression in the adipose tissue, in mice after a long-term (5-month) HFD-induced obesity. For this purpose, we performed miRNA profiling and qPCR validation of our results. The expression of some miRNAs was found either increased or decreased in HFD-fed mice, compared to their SD-fed counterparts. Some of the results are in accordance with previous studies, such as the up-regulation of mmu-miR-221 and mmu-miR222 cluster and the down-regulation of the mmu-miR-200 family, as well as of mmu-miR-204, mmu-miR-30a*, mmu-miR-193, mmu-miR-378 and mmu-miR-30e*. These findings have also been confirmed in other independent studies. Their importance lies in that they suggest new miRNA players in the field of obesity. However, further in vivo and in vitro experimental investigation is warranted in order to describe them as potential pharmaceutical targets.

The rest of the differentially expressed miRNAs, after HFD feeding, has not been previously reported, regarding adipogenesis and obesogenesis. These include on the one hand the up-regulated miRNAs: mmu-miR-342-3p, mmu-miR-142-3p, mmu-miR-142-5p, mmu-miR-21, mmu-miR-335-5p, mmu-miR-146a, mmu-miR-146b, mmu-miR-674 and mmu-miR-379; and on the other hand the down-regulated ones after HFD-induced obesity: mmu-miR-122, mmu-miR-133p, mmu-miR-1, mmu-miR-30a, mmu-miR-192 and mmu-miR-203.

To get a deeper insight into the potential functional significance of the differentially expressed miRNAs in white adipose tissue, their validated target genes were identified using the miRWalk algorithm. Then, Gene Ontology analysis classified the potential enriched functions of these genes in groups. A single miRNA is likely to regulate many genes and each target gene may be regulated by more than one miRNAs, as revealed by the miRWalk algorithm. Therefore, the potential network of miRNAs and genes that are involved in adipose tissue during HFD-induced obesity seems to be highly complicated. Nevertheless, special focus was given to certain highly enriched functions related to obesity and adipogenesis.

Firstly, a significant function of the differentially expressed miRNA targets is cell differentiation and the regulation of cell differentiation (Figs.10, 11). This is linked to the fact that obesity is characterized of both hypertrophy (enlargement of the existing adipocytes) and hyperplasia (adipogenesis or adipocyte differentiation) [28]. It is also important to point out that miRNAs that are induced during adipogenesis, are usually repressed during obesity development [19].

The mmu-miR-142-5p-regulated genes, Bmp-4 and Fgf10, are involved in adipogenesis regulation [29]. Furthermore, Akt1, a target of mmu-miR-142-3p, is also involved in this regulation [30]. Both mmu-miR-142-3p and mmu-miR-142-5p derive from the stem loop precursor of mmu-miR-142. This is the first study to connect mmu-miR-142 with obesity; however, further experiments are warranted to elucidate its role in this process.

Mmu-miR-21 and mmu-miR-146a are also potentially involved in adipocyte differentiation by targeting C/EBP beta [31] and ApoE, respectively [32]. Mmu-miR-146a, which shares 91% homology with mmu-miR-146b in a length of 22 nt [33], is mainly implicated in inflammatory response by being induced by NFκB [34] and by targeting interferon gamma (as revealed by miRWalk in this study). In contrast with mmu-miR-21 for which there are studies describing it to increase during adipocyte differentiation [35], [36], no reference is available regarding mmu-miR-146a, mmu-miR-146b and obesity. Taking into account the up-regulation of mmu-miR-21, mmu-miR-146a and mmu-miR-146b after HFD-induced obesity in the present study, these miRNAs are suggested as a research subject for future studies in obesity.

Mmu-miR-222 also belongs to the miRNA group being highly enriched during adipogenesis. It has already been shown to be up-regulated in obesity and down-regulated in adipogenesis [19], [37]. Hif-1α and TNF-α are two potential mmu-miR-222 targets participating in the regulation of cell differentiation. Both genes have been described to be indirectly implicated in adipogenesis [38], [39].

Mmu-miR-342-3p and mmu-miR-379 were both found to be up-regulated after HFD-induced obesity in the present study. These miRNAs have not been described before with relevance to adipogenesis and obesity. A possible link between mmu-miR-342-3p and obesity is the glucagon that is a potential target of this miRNA and is implicated in obesogenesis [40]. Further studies are needed to clarify any potential role of these miRNAs in these processes.

Next, focus was given on the enriched process “regulation of metabolic process”. Mmu-miR-200b and mmu-miR-200c(members of the mmu-miR-200 family), mmu-miR-203 and mmu-miR-192 target Zeb1 and Zeb2 that regulate epithelial to mesenchymal transition [41] and have recently been implicated in adipogenesis and obesity [42]. The down-regulation of mmu-miR-200b and mmu-miR-200c after HFD-induced obesity, is in accordance with a previous study which showed that the mmu-miR-200 family promotes adipogenesis [43]. The down-regulation of mmu-miR-203 and mmu-miR-192 is described for the first time and warrants further elucidation.

Of the down-regulated miRNAs after HFD-induced obesity, the putative targets of mmu-miR-1, mmu-miR-204 and mmu-miR-133b, are implicated in cell differentiation. Mmu-miR-1 has mainly been characterized as a muscle-specific miRNA [44] and it has been found to be expressed in brown adipocytes [45]. This is in accordance with the theory that brown adipocytes and muscle share common precursor cells. Mmu-miR-1 targets UCP-1, which uncouples oxidative phosphorylation from the production of ATP in brown adipocytes and energy is dissipated as heat [46]. The expression of mmu-miR-1 that we detected in white adipose tissue, can be due to the presence of brown adipocytes in white adipose tissue [47] and may be important in the metabolic adaptation that occurs in the mouse during HFD-feeding. Mmu-miR-204 and mmu-miR-133b target Runx2 which promotes osteogenesis and represses adipogenesis [48]. Mmu-miR-204 has already been described to promote adipogenesis and repress osteogenesis through Runx2 repression [49].

The down-regulation of mmu-miR-30a*, mmu-miR-30e*, mmu-miR-193 and mmu-miR-378 during HFD-induced obesity is consistent with previous studies [19], [37], [50]. This indicates that these miRNAs should be further studied, so as to check if they can successfully be targets for obesity treatment. On the other hand, the down-regulation of mmu-miR-122 and mmu-miR-130a during HFD-induced obesity that we report has not been described before. Mmu-miR-122 is mainly expressed in the liver and makes up for the 70% of all the liver miRNAs [51]. It is known to be implicated in cholesterol biosynthetic pathway and fatty acid metabolism [52]. Its expression in adipose tissue has been found to be 200-fold less than in liver [53] and further characterization of its potential role in obesogenesis is warranted. Similarly, mmu-miR-130a is known to be a pro-angiogenic miRNA and its role in adipose tissue has not been described before [54]. Taking these data into account, it seems that miRNAs play a regulatory role in the development of obesity. The present study confirms the results of previous reports with reference to the differential expression of miRNAs, after HFD feeding and during adipogenesis. Moreover, we present novel miRNAs which are involved in the aforementioned processes. Their exact role remains to be further investigated, so as to come to a conclusion regarding their usefulness as therapeutical targets for the prevention and treatment of obesity, diabetes type 2 and metabolic syndrome.

## Supporting Information

Figure S1 **Densities of individual-channel intensities for two-color microarray data for all slides.** The density plot shows the effect of the normalization as the signal distribution of the two channels is more similar after normalization than before. (Hy3; green, Hy5; red).
(TIF)

Figure S2 **Box plots illustrating the distribution of signals measured on the capture probes before and after normalization.** The box-plots show that the majority of capture probes have similar signals in both channels suggesting that the miRNAs have similar expression levels in the paired samples on each slide (Capture probes with a log_2_ median ratio of “0” on the Y-axis correspond to miRNAs that are equally expressed in the 2 different samples on this slide). The lower boundary of the box indicates the 25^th^ percentile, the line within the box shows the median, and the upper edge of the box marks the 75^th^ percentile. Whiskers above and below each box indicate the 95^th^ and 5^th^ percentiles. All data points that lie outside the 5^th^ and 95^th^ percentiles are shown as symbols.
(TIF)

Figure S3 **The positive effect from normalization is illustrated on each slide with an M-A plot before and after normalization.** After normalization the spots appear symmetrically scattered around the horizontal line M = 0. The difference between the two channels (M) is now independent of the average intensity level of the two channels. (slide 1-control 1, A; control 2-sample 2, B; slide 3-sample, C)
(TIF)

Figure S4 **Gene stability measurement (M) for endogenous control genes.** For every endogenous control gene, the pair-wise variation with all other endogenous controls was determined as a gene stability measurement M. An M value below 1.5 was recommended and genes with expression stability above 1.5 were considered unstable across the samples and unsuitable for endogenous controls in this experiment. U6 snRNA, mmu-191, mmu-423-5p, mmu-361 and mmu-103 were considered acceptable and used for normalization. The gene 5S rRNA was unstable across the samples and therefore excluded from the normalization.
(TIF)

Figure S5 **Weight measurements of mice before and after HFD feeding (5 months).** Wild type mice were fed a SD or a HFD diet for 5 months. At the beginning of the experiment the two groups of mice had no difference in their weights. After 5 months on SD or HFD, the mice of the HFD group were significantly heavier than those of the SD group, as expected. Data show mean±SEM values. N = 8 for each diet type. *p<0.0001. SD; standard diet, HFD; high-fat diet.
(TIF)

Figure S6 **Wild type mice fed a HFD for 5 months become more insulin resistant and less glucose tolerant than the SD-fed ones.** Results from glucose and insulin tolerance tests are shown at the beginning of the experiment (day 0) and after 5 months on SD or HFD feeding. Data show means±SEM. N = 8 for each diet type. *p<0.01, **p<0.001. IPGTT: intraperitoneal glucose tolerance test, IPITT: intraperitoneal insulin tolerance test. SD; standard diet, HFD; high-fat diet.
(TIF)

Table S1 **Upregulated and downregulated miRNAs with their corresponding validated target genes based on miRWalk algorithms**
(XLS)

Table S2 **Target genes of the upregulated miRNAs and the enriched processes in which they participate.**
(XLS)

Table S3 **Target genes of the downregulated miRNAs and the enriched processes in which they participate.**
(XLS)
