# Supplementary figures and images for: Differential Expression of MicroRNAs in Adipose Tissue after Long-Term High-Fat Diet-Induced Obesity in Mice
Source: PLoS One. 2012 Apr 4;7(4):e34872. doi: 10.1371/journal.pone.0034872 (PMC3319598; doi:10.1371/journal.pone.0034872)

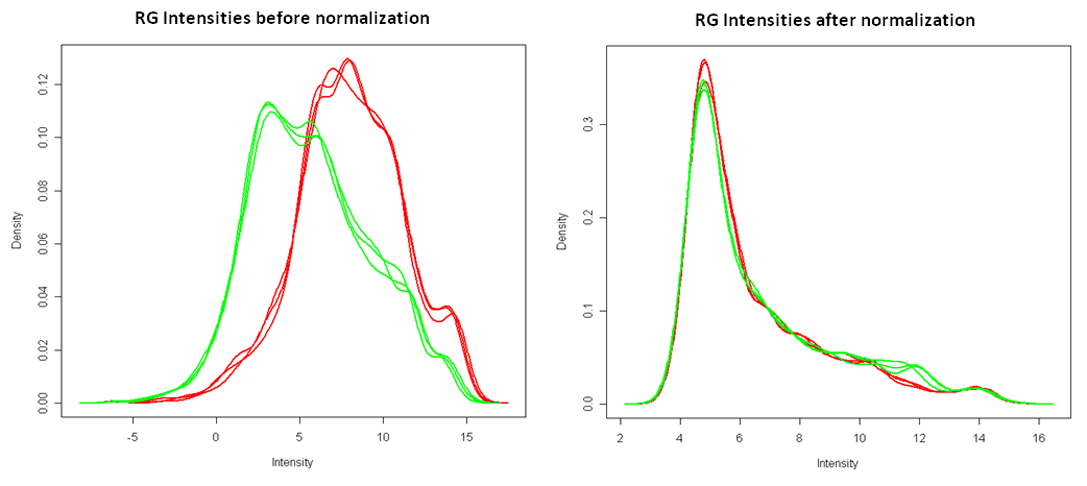

Supplement: Figure S1 — Densities of individual-channel intensities for two-color microarray data for all slides. The density plot shows the effect of the normalization as the signal distribution of the two channels is more similar after normalization than before. (Hy3; green, Hy5; red). (TIF) [file pone.0034872.s001.tif]

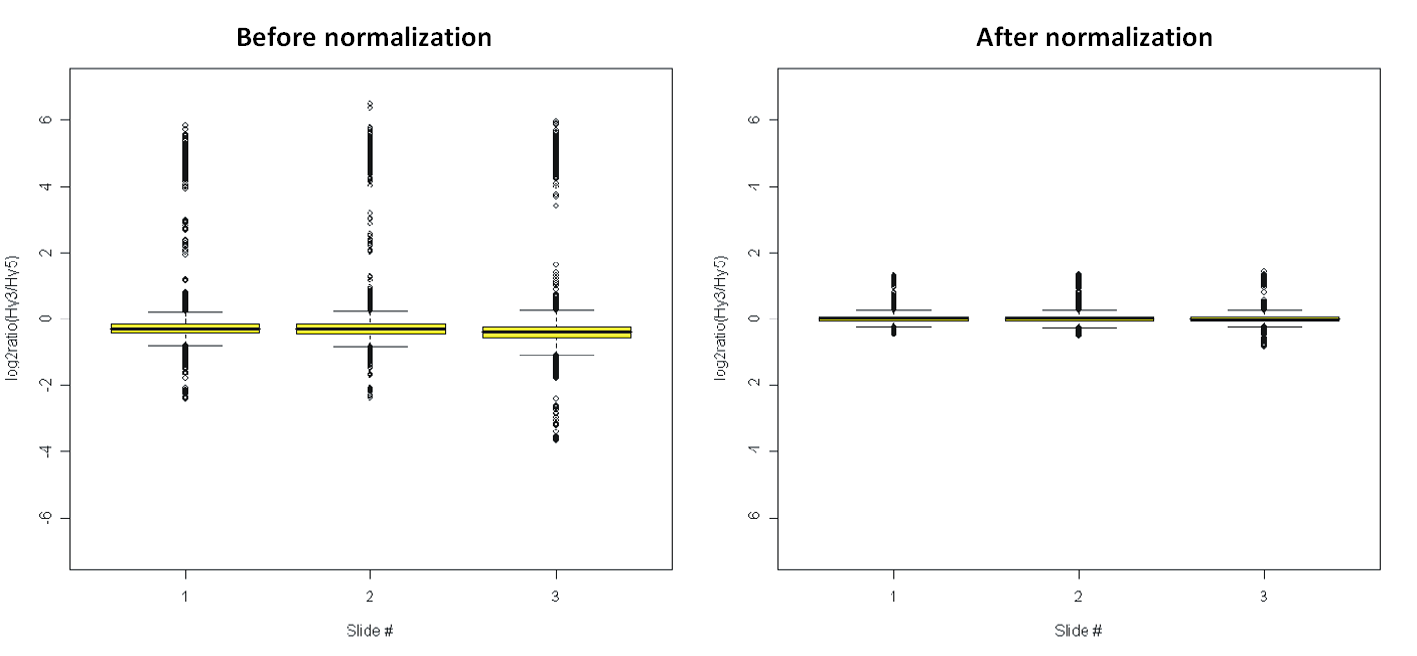

Supplement: Figure S2 — Box plots illustrating the distribution of signals measured on the capture probes before and after normalization. The box-plots show that the majority of capture probes have similar signals in both channels suggesting that the miRNAs have similar expression levels in the paired samples on each slide (Capture probes with a log2 median ratio of “0” on the Y-axis correspond to miRNAs that are equally expressed in the 2 different samples on this slide). The lower boundary of the box indicates the 25th percentile, the line within the box shows the median, and the upper edge of the box marks the 75th percentile. Whiskers above and below each box indicate the 95th and 5th percentiles. All data points that lie outside the 5th and 95th percentiles are shown as symbols. (TIF) [file pone.0034872.s002.tif]

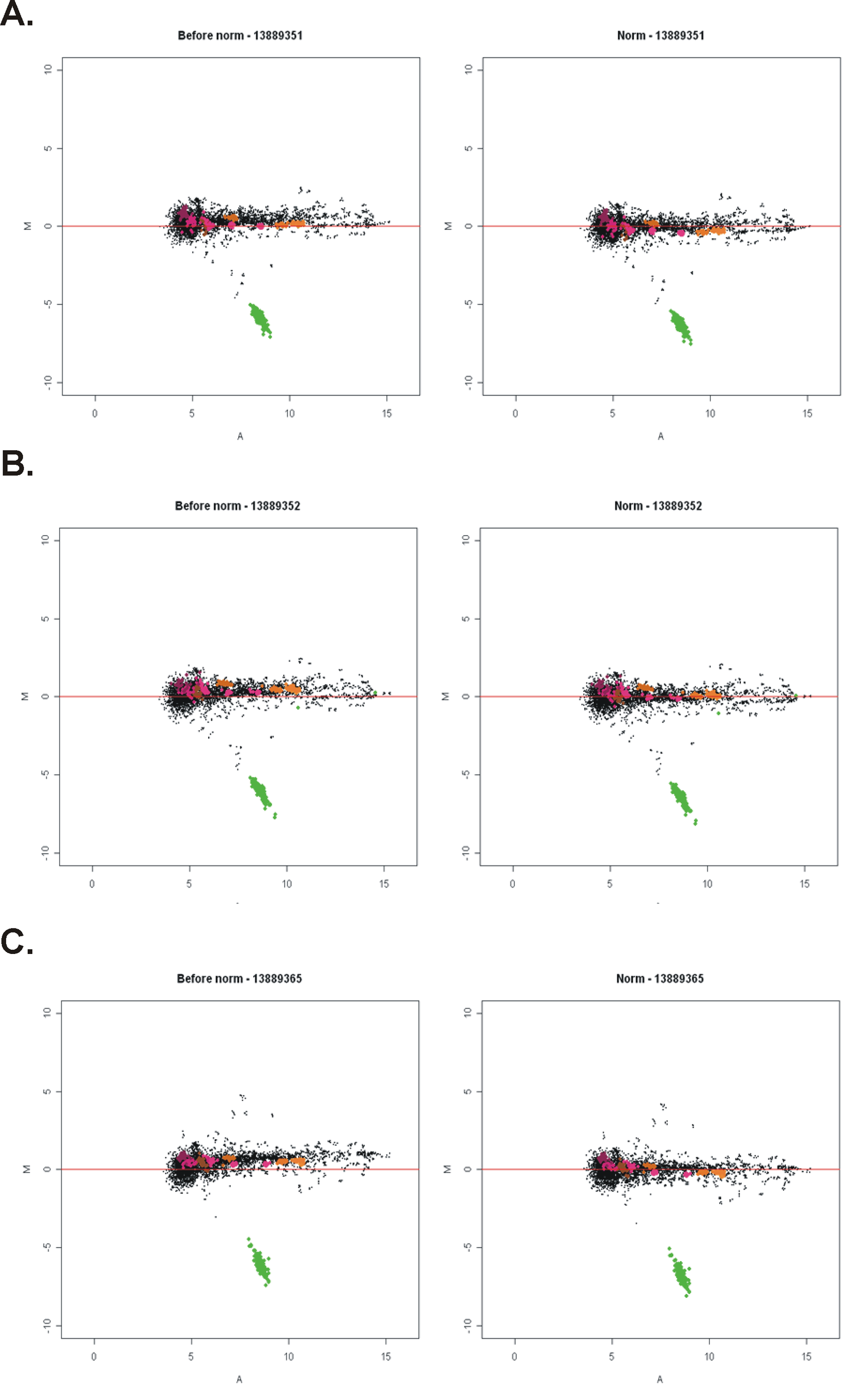

Supplement: Figure S3 — The positive effect from normalization is illustrated on each slide with an M-A plot before and after normalization. After normalization the spots appear symmetrically scattered around the horizontal line M = 0. The difference between the two channels (M) is now independent of the average intensity level of the two channels. (slide 1-control 1, A; control 2-sample 2, B; slide 3-sample, C) (TIF) [file pone.0034872.s003.tif]

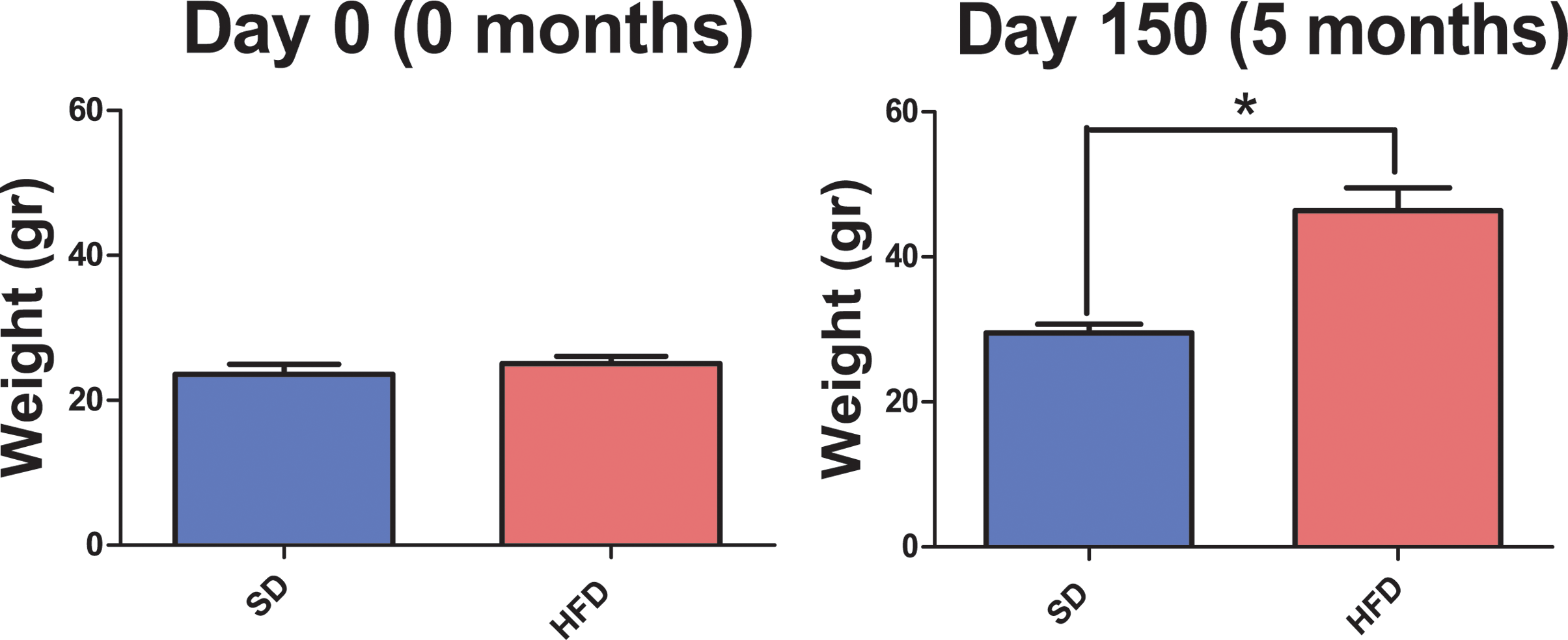

Supplement: Figure S5 — Weight measurements of mice before and after HFD feeding (5 months). Wild type mice were fed a SD or a HFD diet for 5 months. At the beginning of the experiment the two groups of mice had no difference in their weights. After 5 months on SD or HFD, the mice of the HFD group were significantly heavier than those of the SD group, as expected. Data show mean±SEM values. N = 8 for each diet type. *p<0.0001. SD; standard diet, HFD; high-fat diet. (TIF) [file pone.0034872.s005.tif]

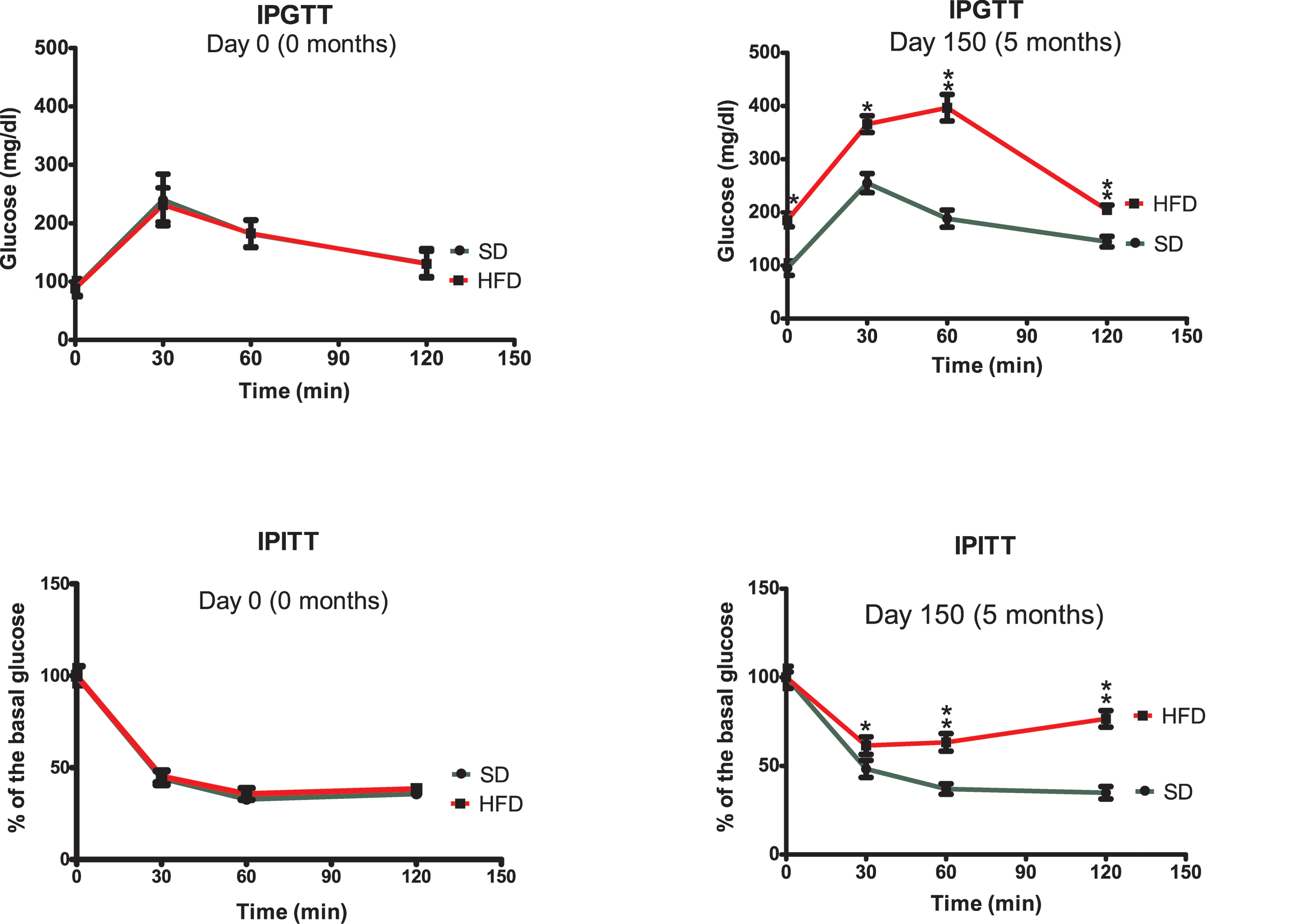

Supplement: Figure S6 — Wild type mice fed a HFD for 5 months become more insulin resistant and less glucose tolerant than the SD-fed ones. Results from glucose and insulin tolerance tests are shown at the beginning of the experiment (day 0) and after 5 months on SD or HFD feeding. Data show means±SEM. N = 8 for each diet type. *p<0.01, **p<0.001. IPGTT: intraperitoneal glucose tolerance test, IPITT: intraperitoneal insulin tolerance test. SD; standard diet, HFD; high-fat diet. (TIF) [file pone.0034872.s006.tif]
